# Supplementary material for: Subcutaneous Emphysema Related to Dental Treatment: A Case Series
Source: Healthcare (Basel). 2022 Feb 1;10(2):290. doi: 10.3390/healthcare10020290 (PMC8872011; doi:10.3390/healthcare10020290)
Supplement: Supplementary file 1 [file healthcare-10-00290-s001.zip › healthcare-1521018-supplementary.pdf]

Supplementary Materials: Measurement method of subcutaneous emphysema using 3D simulation from CT data

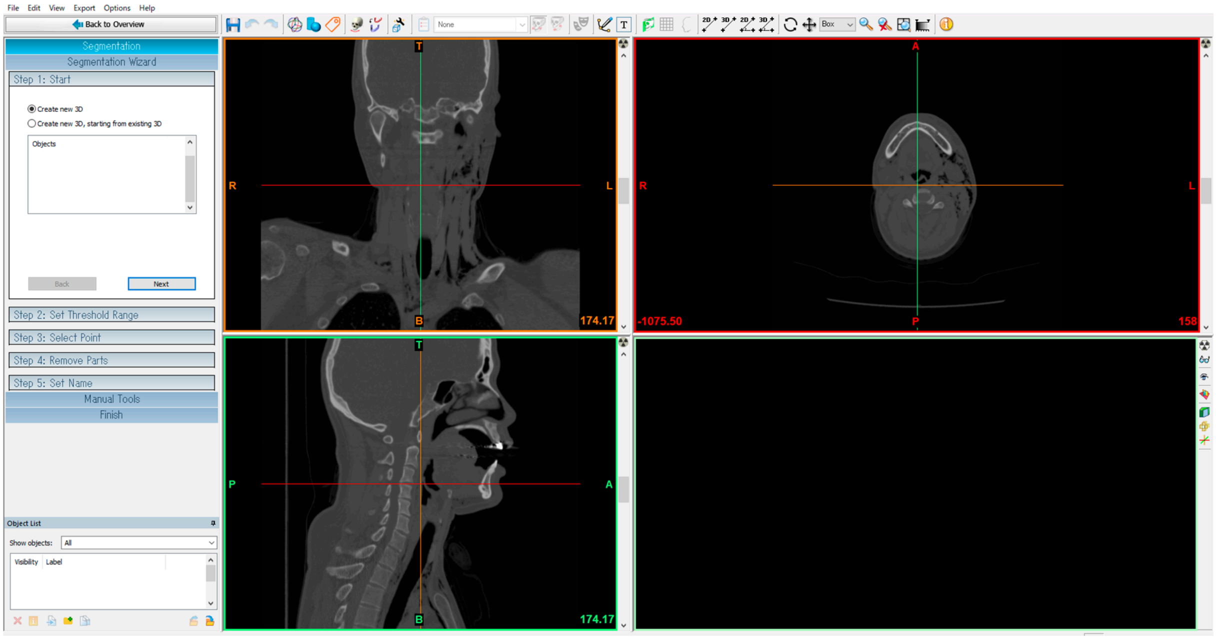

1) Reading CT data (DICOM): Save the DICOM data output during CT shooting to the PC on which ProPlan CMF 3.0 is installed, start the software, and open the data.

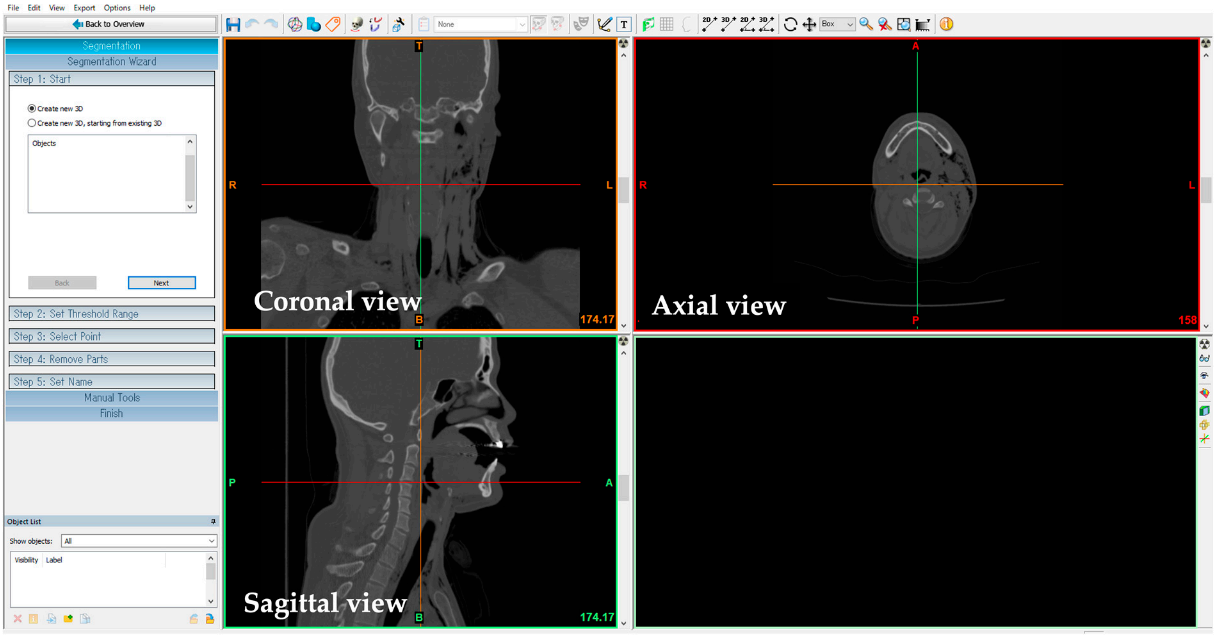

2) Setting the threshold range: Select bone level range between 226–3071.

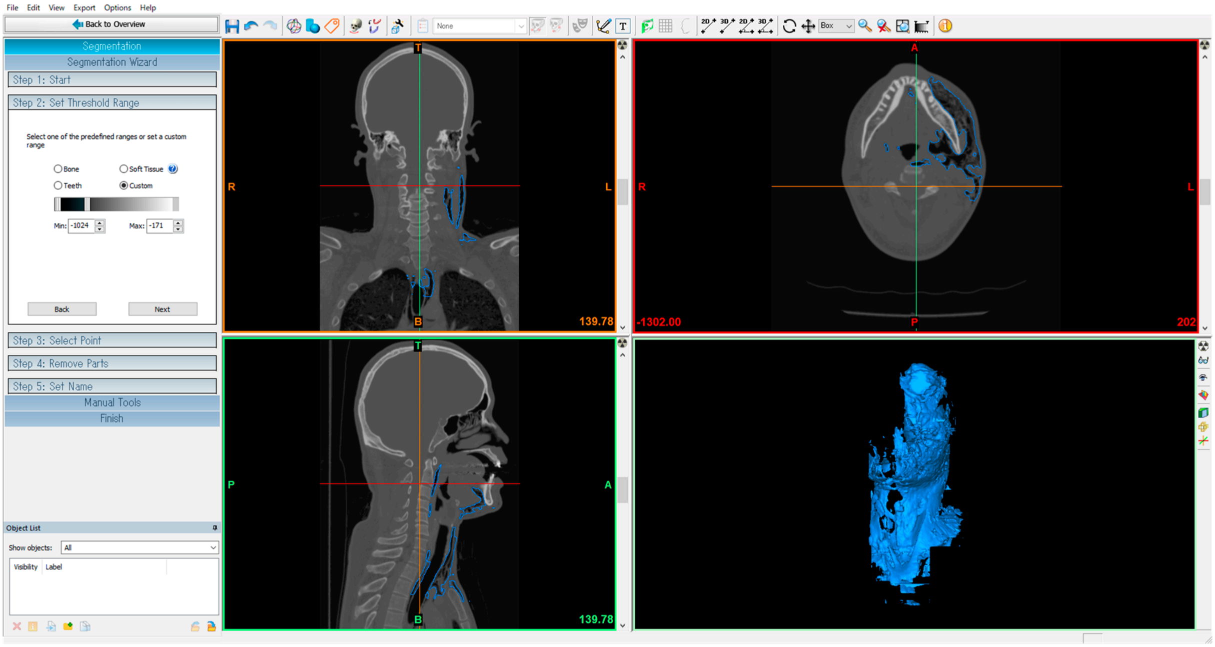

3) Setting the threshold range: Set a custom air range between -1024 – -170.

4) Remove parts: Select the regions that have to be removed in 2D or 3D. If you have selected a site different from subcutaneous emphysema, such as the airway, we removed it manually.

5) Segment the 3D objects that we need for analysis.

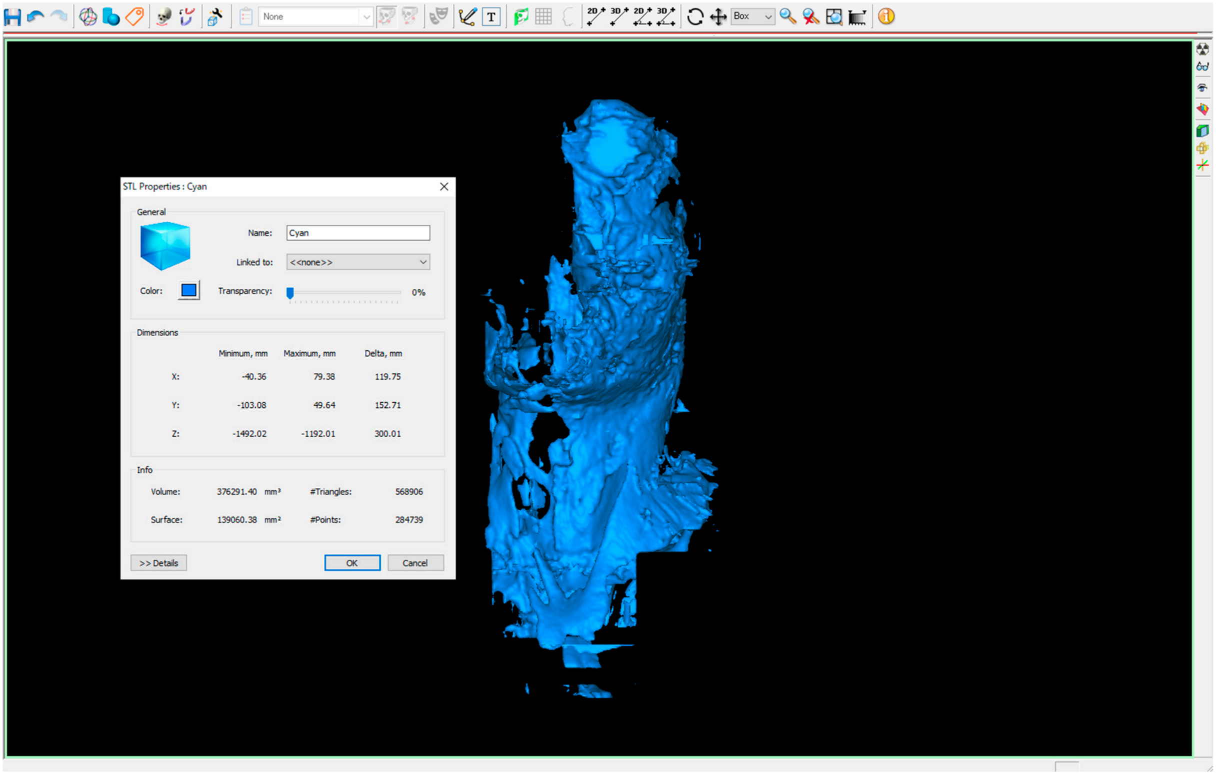

6)Analyze the emphysema volume: The 3D image was converted to stereolitho-graph format, and the volume was measured using xyz coordinates.
